# *Pseudomonas putida* mt-2 tolerates reactive oxygen species generated during matric stress by inducing a major oxidative defense response

**DOI:** 10.1186/s12866-015-0542-1

**Published:** 2015-10-06

**Authors:** Nanna B. Svenningsen, Danilo Pérez-Pantoja, Pablo I. Nikel, Mette H. Nicolaisen, Víctor de Lorenzo, Ole Nybroe

**Affiliations:** Department of Plant and Environmental Sciences, Section of Genetics and Microbiology, University of Copenhagen, Thorvaldsensvej 40, 1871 Frederiksberg C, Denmark; Systems and Synthetic Biology Program, Centro Nacional de Biotecnología (CNB-CSIC), C/ Darwin 3, 28049 Madrid, Spain

**Keywords:** *Pseudomonas putida* mt-2, Matric stress, ROS, Oxidative stress, Bioreporters

## Abstract

**Background:**

Soil bacteria typically thrive in water-limited habitats that cause an inherent matric stress to the cognate cells. Matric stress gives rise to accumulation of intracellular reactive oxygen species (ROS), which in turn may induce oxidative stress, and even promote mutagenesis. However, little is known about the impact of ROS induced by water limitation on bacteria performing important processes as pollutant biodegradation in the environment. We have rigorously examined the physiological consequences of the rise of intracellular ROS caused by matric stress for the toluene- and xylene-degrading soil bacterium *Pseudomonas putida* mt-2.

**Methods:**

For the current experiments, controlled matric potential stress was delivered to *P. putida* cells by addition of polyethylene glycol to liquid cultures, and ROS formation in individual cells monitored by a specific dye. The physiological response to ROS was then quantified by both RT-qPCR of RNA transcripts from genes accredited as proxies of oxidative stress and the SOS response along with cognate transcriptional GFP fusions to the promoters of the same genes.

**Results:**

Extensive matric stress at −1.5 MPa clearly increased intracellular accumulation of ROS. The expression of the two major oxidative defense genes *katA* and *ahpC,* as well as the hydroperoxide resistance gene *osmC*, was induced under matric stress. Different induction profiles of the reporters were related to the severity of the stress. To determine if matric stress lead to induction of the SOS-response, we constructed a DNA damage-inducible bioreporter based on the LexA-controlled phage promoter P_PP3901_. According to bioreporter analysis, this gene was expressed during extensive matric stress. Despite this DNA-damage mediated gene induction, we observed no increase in the mutation frequency as monitored by emergence of rifampicin-resistant colonies.

**Conclusions:**

Under conditions of extensive matric stress, we observed a direct link between matric stress, ROS formation, induction of ROS-detoxifying functions and (partial) activation of the SOS system. However, such a stress-response regime did not translate into a general DNA mutagenesis status. Taken together, the data suggest that *P. putida* mt-2 can cope with this archetypal environmental stress while preserving genome stability, a quality that strengthens the status of this bacterium for biotechnological purposes.

## Background

The impact of environmental factors on the survival and activity of microorganisms involved in pollutant biodegradation is of considerable interest, as environmental stress may represent a bottleneck for their optimal performance in natural environments. Hence, previous investigations have addressed the significance of various environmental stressors (e.g., variable nitrogen sources, oxidative stressors, as well as carbon and iron starvation) on transcriptional profiles of catabolic genes in *Pseudomonas putida* mt-2 [1]. This toluene- and xylene-degrading soil bacterium, carrying the catabolic TOL plasmid pWW0, as well as its plasmid-cured derivative *P. putida* KT2440, are well studied paradigm organisms for applications in environmental biotechnology [2–5].

For bacterial cells residing in unsaturated habitats such as surface soils, fluctuation in water availability represents a major environmental factor affecting their survival and activity [6, 7]. In non-saline soils capillary forces and physical sorption of water to solids, together constituting the soil matric potential, are the dominating factors determining water availability [8]. Low matric potentials (i.e., desiccation) limit transport and diffusion of nutrients, impair microbial mobility, and negatively affect the physiological activity of soil bacteria [9–11]. Importantly, desiccation may increase the endogenous formation of reactive oxygen species (ROS), and a single study has shown that *P. putida* micro-colonies growing on a medium containing polyethylene glycol with molecular weight of 8000 (PEG-8000), used to simulate matric stress, accumulate more ROS than the corresponding micro-colonies formed under water-replete conditions [12]. However, the physiological consequences of ROS accumulation are currently not known in detail.

In *Pseudomonas* and other bacteria protection against ROS and its harmful effects involves different strategies to maintain the amount of oxidants at a non-toxic level, and to repair cellular damage caused by increased levels of ROS [13, 14]. Small antioxidant molecules, for instance reduced glutathione and thioredoxin, and redirection of metabolic pathways towards routes that regenerate reducing power (e.g., NADPH) play an important role in the removal of ROS [15–18]. However, inducible enzymes, such as superoxide dismutases, catalases, and peroxidases, seem to constitute the major component of the bacterial oxidative stress defense system [19, 20]. Surprisingly, whole genome transcriptome studies and screens for desiccation-induced genes in *P. putida* show upregulation of quite few oxidative stress responders in cells subjected to matric stress [21, 22]. Hence, the expression of the major defense genes as *katA* (PP0481, catalase), *ahpCF* (PP2439-PP2440, alkylhydroperoxide reductase), or *sodAB* (PP0946 and PP0915, superoxide dismutase), was not induced in these studies. This might suggest that matric stress is not a very strong inducer of these oxidative stress defense genes, and that cells are either not well protected against the consequences of increased ROS levels, or use other defense mechanisms against matric stress-mediated accumulation of ROS.

If imbalances occur between the oxidant accumulation and the protective capacity of the defense system, the desiccated cells experience oxidative stress, a condition where ROS may reach levels that generate damage on the DNA [23]. DNA damage induces SOS-response dependent error-prone DNA polymerases, eventually leading to accumulation of mutations [24, 25]. Oxidative damage to DNA is an important source of genetic variations in stressful environments [26–28]. Nevertheless, it has not been determined whether matric stress actually induces the SOS response, i.e., the LexA1 and LexA2 regulons in *P. putida*, and whether such an induction could result in increased genetic and phenotypic diversity of the surviving bacteria, and thereby promote adaptation to the stressful conditions bacteria encounter in unsaturated environments [12, 29].

The overall objective of this study was to obtain a comprehensive understanding of how *P. putida* mt-2 responds to increased ROS levels generated during matric stress. We carried out our experiments in a completely mixed and homogenous pure culture system, and used PEG-8000 to lower external water potential and hence simulate matric stress [8, 30]. Our first specific aim was to determine whether major oxidative stress defense genes were differentially expressed under matric stress. Most studies on defense responses in desiccated bacterial cells rely on bulk measurements of gene expression (see for instance [21, 31]). However, in cases where a gene is not highly expressed there is a risk of masking the signal by the bulk measurement [32]. In addition, the response to environmental stimuli among individual cells of isogenic populations is often heterogeneously distributed, frequently with minor subpopulations dominating the overall population behavior [33–35]. Therefore, we developed a panel of fluorescent whole-cell bioreporters to determine oxidative defense gene expression in *P. putida* mt-2 at the single cell level through flow cytometry. Our final specific aim was to determine if the *P. putida* SOS-response was activated during matric stress, and if such activation resulted in increased mutations rates. For that purpose, we constructed a bioreporter based on a LexA1/LexA2-regulated phage promoter [25], and determined if matric stress altered the frequency of mutations leading to resistance towards rifampicin.

## Results

### Matric stress induces production of endogenous ROS

**Fig. 1:**
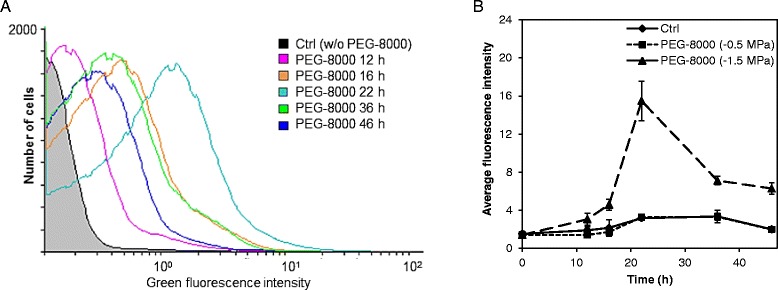
Accumulation of reactive oxygen species (ROS) in *P. putida* mt-2 following matric stress. Matric stress was simulated by incubation in liquid culture media amended with PEG-8000 to obtain matric potentials of −0.5 MPa or −1.5 MPa. ROS were detected as oxidation-dependent green fluorescence of the ROS-sensitive probe 2′,7′-dichlorodihydrofluorescein diacetate. **a** Histogram illustrating the fluorescence from one representative experiment where cells were either incubated without PEG-8000 (considered control, Ctrl) or exposed to PEG-8000 (−1.5 MPa). **b** Average fluorescence intensity of cells from PEG-8000 treated cultures and Ctrl cultures. Data represent mean values ± S.D. (*n* = 3)

In order to simulate matric stress of −0.5 MPa or a more extensive matric stress of −1.5 MPa, the so-called permanent plant wilting point (referred to as “mild” or “extensive” matric stress, respectively) we grew wild-type *P. putida* mt-2 cells in liquid media containing the non-permeating solute PEG-8000 to lower the water potential [8]. The formation of intracellular ROS was detected as the fluorescence arising from the ROS-sensitive green fluorescent dye 2′,7′-dichlorodihydrofluorescein diacetate (H_2_DCF-DA) by flow cytometry. Accumulation of ROS was not detectable in cells incubated under conditions of mild matric stress (−0.5 MPa, Fig. 1). Reduction of the matric potential to −1.5 MPa on the other hand clearly increased the endogenous ROS formation as compared to the untreated control (Fig. 1). The response was pronounced after >10 h and accumulation of ROS peaked after 22 h (~15 fold increase in ROS-dependent fluorescence compared to control cells). These results documented that cells experiencing extensive matric stress even in liquid culture accumulate ROS, and prompted us to construct a panel of bioreporters for oxidative stress responses.

### Construction of bioreporters for oxidative stress

**Fig. 2:**
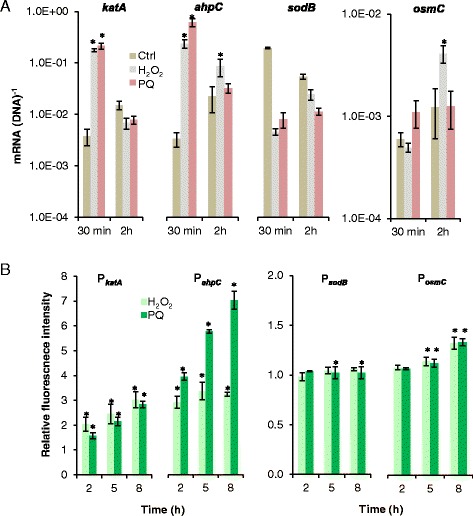
Response of oxidative stress-associated genes and promoters. **a** Expression of *katA*, *ahpC, sodB*, and *osmC* from *P. putida* mt-2 in the presence of H_2_O_2_ (1.5 mM), paraquat (PQ, 100 μM), or without an oxidative stress inducer (control, Ctrl) as measured by RT-qPCR. Cells were sampled 30 min and 2 h after addition of the stress inducer. Data are mean values ± S.D. (*n *= 3) of mRNA per DNA of the gene in question. Asterics (*) indicate mean values of treated cultures that are significantly (*P *< 0.05) higher than mean values from Ctrl cultures. Note the different scale for the *y*-axis of the diagram of *osmC* expression. **b** Induction of pSEVA237M-based bioreporters in *P. putida* mt-2 cultured in the presence of H_2_O_2_ (1.5 mM) or PQ (100 μM) after 2–8 h of stress exposure. Data are mean values ± S.D. (*n* = 3) of average fluorescence intensity of cells treated with either H_2_O_2_ or PQ normalized to the average fluorescence intensity of Ctrl cells. Asterisks (*) indicate mean values from treated cultures that are significantly (*P* < 0.05) higher than mean values from Ctrl cultures. Note that values on the *y*-axis of the diagram of P_*katA*_ and P_*ahpC*_ are different from the values in the diagram of P_*sodB*_ and P_*osmC*_

We constructed four oxidative stress responsive bioreporters based on the promoters for the major stress defense genes *katA* (PP0481), *ahpC* (PP2439), *sodB* (PP0915), and further included *osmC* (PP0089) as the expression of this gene appears to be upregulated in surface-grown *P. putida* cells exposed to matric stress imposed by suction [21]. These four bioreporters were based on the medium-copy-number plasmid vector, pSEVA237M of the SEVA collection [36]. Initially we quantified the expression of the selected genes by RT-qPCR under conditions that are known to increase the levels of intracellular ROS. In cells incubated with 1.5 mM H_2_O_2_, both *katA* and *ahpC* were transiently upregulated after 30 min (Fig. 2a). A comparable or even higher expression level was observed for cells treated with the superoxide-generator, paraquat (PQ, added at 100 μM). Also the *osmC* gene was significantly (*P* < 0.05), though not very highly, induced in cells treated with H_2_O_2,_ but not with PQ, and the response of *osmC* to oxidative stress was slower than the responses of *katA* and *ahpC*. Upregulation of *sodB* was on the other hand not detectable with any of the tested oxidative stress inducers, neither after 30 min nor after 2 h of incubation. On the contrary, expression of *sodB* seemed to be downregulated in the stressor-treated cells.

The ability of the four plasmid-based bioreporters to respond to the oxidative stress conditions created by incubation with 1.5 mM H_2_O_2_ or 100 μM PQ was tested by flow cytometry. Figure 2b illustrates the average fluorescence intensity of H_2_O_2_- or PQ-treated cells normalized to the average fluorescence intensity of control cells incubated without a stress inducer. In contrast to the transient upregulation of the *katA* and *ahpC* after 30 min seen by RT-qPCR, the signal from the reporters having msf-*gfp* fused to P_*katA*_ or P_*ahpC*_ increased up to at least 8 h of stress exposure. The reporter based on P_*osmC*_ had a weaker and later response to H_2_O_2_ and PQ than P_*katA*_ and P_*ahpC*_, which is in agreement with the profile of *osmC* expression measured by RT-qPCR (compare Fig. 2a and b). However, in contrast to the RT-qPCR data from the PQ treatment, flow cytometry data for this treatment revealed a small, but significant (*P* < 0.05) induction of P_*osmC*_, emphasizing the improved sensitivity of the reporters based on detection of fluorescence of the stable GFP protein. The reporter based on *sodB* did not respond to PQ; however, after 5 and 8 h there was a minor induction of P_*sodB*_ in cells exposed to H_2_O_2_ (*P* < 0.05).

### Matric stress activates major oxidative stress response genes

**Fig. 3:**
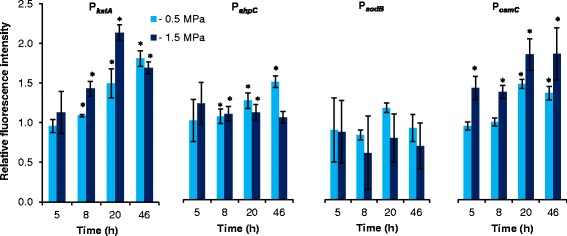
Induction of pSEVA237M-based bioreporters in *P. putida* mt-2 under matric stress. Bioreporter cells were exposed to mild matric stress of −0.5 MPa or extensive matric stress of −1.5 MPa, and fluorescence was detected by flow cytometry. Data are mean values ± S.D. (*n* = 3) of average fluorescence intensity of cells incubated under matric stress of −0.5 MPa or −1.5 MPa normalized to the average fluorescence intensity of Ctrl cells. Asterisks (*) indicate mean values from treated cultures that are significantly (*P* < 0.05) higher than mean values from Ctrl cultures.

The reporter strains were used to evaluate the physiological response to ROS accumulation under matric stress. As shown in Fig. 3, the *katA*, *ahpC*, and *osmC* reporters all responded to the two tested matric stress scenarios. In contrast, the *sodB* promoter was not activated by matric stress. The induction profiles of P_*katA*_, P_*ahpC*_, and P_*osmC*_ differed noticeable as compared to induction profiles obtained for cells exposed to H_2_O_2_ or PQ (compare Figs. 2b and 3). At the time points tested here, the *katA* reporter showed the best response under both matric stresses. However, even the P_*osmC*_ reporter that only showed a weak induction by H_2_O_2_ and PQ was induced to an extent comparable to P_*katA*_ under extensive matrix stress (−1.5 MPa). Despite lack of detection of ROS accumulation in cells exposed to mild matric stress of −0.5 MPa (Fig. 1), P_*katA*_*,* P_*osmC*_ and P_*ahpC*_ were induced under this stress scenario. After 46 h the induction of P_*ahpC*_ was even higher for cells exposed to mild matric stress compared to cells incubated under extensive matric stress (−1.5 MPa). Hence, we could demonstrate induction of two major oxidative defense genes, as well as of *osmC*, during matric stress, and relate different induction profiles of the different reporters to the severity of the stress.

### Matric stress induces the LexA-regulated PP3901 gene, but does not increase mutation frequency

**Fig. 4:**
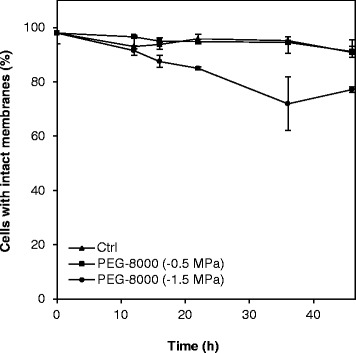
Viability of *P. putida* mt-2 cells exposed to matric stress. The viability, following incubation without matric stress (control, Ctrl), under mild matric stress (−0.5 MPa), or under extensive matric stress (−1.5 MPa), was determined by live/dead staining and flow cytometry. Data are mean values and error bars represent the standard deviation of three biological replicate samples

If intracellular levels of ROS exceed the cellular defense capacity, oxidative stress is encountered which may lead to DNA-damage and ultimately cell death. To determine if matric stress resulted in enhanced cell death, a live/dead viability staining was performed and analyzed by flow cytometry. In accordance with the lack of detection of ROS accumulation under mild matric stress, cell viability was not affected under this condition (Fig. 4). Extensive matric stress did increase the proportion of cells with injured membranes with time of incubation. As the levels of ROS were severe enough to induce an oxidative stress response in the cells, we then speculated that another consequence of these ROS could be to elicit damage to DNA that would induce the SOS-system and eventually translate into an increase of non-lethal mutations in the matric stressed cells.

**Fig. 5:**
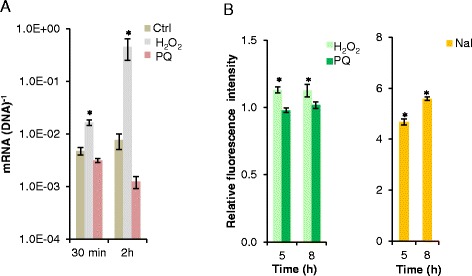
Verification of expression of gene PP3901 induced by oxidative stress conditions. **a** Expression of PP3901 in the presence of H_2_O_2_ (1.5 mM), paraquat (PQ, 100 μM), or without an oxidative stress inducer (control, Ctrl) quantified by RT-qPCR.Data are mean values ± S.D. (*n *= 3) of mRNA per DNA of the gene in question. Asterics (*) indicate mean values of treated cultures that are significantly (*P* < 0.05) higher than mean values from Ctrl cultures. **b** Induction of the bioreporter harboring a transcriptional fusion between PP3901 and msf-*gfp* in *P. putida* mt-2 after exposure 1.5 mM H_2_O_2_, 100 μM PQ (left panel), or 30 μg mL^−1^ nalidixc acid (right panel). Note the different scales on the *y*-axis of the diagrams. Data are mean values of average fluorescence intensity from triplicate cultures ± S.D. Asterisks (*) indicate mean values from treated cultures that are significantly (*P* < 0.05) higher than mean values from Ctrl cultures

A previous study by Abella and colleagues [25] indicated that the gene PP3901 from a resident prophage is required for DNA damage-mediated induction of a number of other prophages in *P. putida*. The PP3901 gene is member of the LexA1 as well as the LexA2 regulons; hence, we exploited the promoter of this gene for the construction of a DNA damage-inducible bioreporter. According to RT-qPCR analysis, expression of PP3901 was upregulated in the presence of 1.5 mM H_2_O_2_, while incubation with 100 μM PQ did not increase the expression (Fig. 5a). Consistently, the constructed Tn*7*-based bioreporter carrying a chromosomal integrated P_PP3901_-*gfp* fusion only responded to the H_2_O_2_-treatment, and not to PQ in the concentration tested here (Fig. 5b). The induction of the reporter by H_2_O_2_ was nevertheless low when compared to the induction by the DNA-damaging agent nalidixic acid (Fig. 5b).

**Fig. 6:**
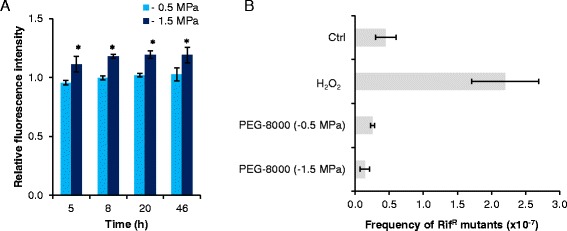
Induction of the DNA-damage inducible P_PP3901_ bioreporter and frequency of mutations conferring rifampicin resistance in *P. putida* mt-2 following exposure to matric stress. **a** Induction of a chromosomal P_*PP3901*_-*gfp* fusion in *P. putida* mt-2 exposed to mild matric stress of −0.5 MPa or extensive matric stress of −1.5 MPa 5–46 h post stress induction. Data represent average fluorescence intensity from cells exposed to matric stress normalized to average fluorescence intensity from control cells.**b** Occurrence of clones conferring resistance to rifampicin (100 μg mL^−1^) normalized to the number of CFU on LB agar without rifampicin following a 72-h incubation with either H_2_O_2_, matric stress (−0.5 MPa or −1.5 MPa), or without any stress inducer (control, Ctrl). Data are mean values ± S.D. (*n* = 3). Asterisks (*) indicate mean values from treated cultures that are significantly (*P* < 0.05) higher than mean values from Ctrl cultures

Subsequently, we analyzed the response of the bioreporter when cells were exposed to matric stress. Mild matric stress did not have any impact, whereas the extensive matric stress resulted in weak, though significant (*P* < 0.05) induction of the bioreporter (Fig. 6a). Despite this DNA-damage mediated gene induction, the extensive matric stress did not increase the mutation frequency as measured by the standard assay of appearance of colonies having resistance to rifampicin (Rif^R^) from cultures incubated under the stress condition (Fig. 6b).

## Discussion

In the current study we address the impact of desiccation, which is considered to be a major stressor in soil environments, on the soil bacterium *P. putida* mt-2 that is widely used as a model organism for studies in environmental microbiology and biotechnology. In particular, we focus on the ability of matric stress to increase intracellular ROS levels and on the physiological responses that *P. putida* launches in response to this stressor.

We have shown that *P. putida* mt-2 cells in liquid cultures accumulate ROS when they are subjected to matric stress simulated by addition of the non-permeating solute PEG-8000 to the culture media. For comparison, Chang and coworkers [12] studied the accumulation of ROS in micro-colony forming cells exposed to a wider range of matric potentials. They showed that ROS accumulation was taking place at water potentials below −0.5 MPa; conditions where cells respond to desiccation by altering the composition of their membrane fatty acids and increase production of the matrix of extracellular polymeric substance, and where physiological constraints affect their activity [11, 21, 37, 38]. The temporal dynamics of ROS accumulation observed by Chang and coworkers [12] differed slightly from our observations. For a matric potential of −1.5 MPa, accumulation of ROS in the surface-grown cells increased slowly to peak after 3 d in their study. The earlier response in our liquid cultures is likely attributable to the different experimental set-up.

Increased oxidative stress under desiccation has been described in other bacterial species, and also in yeast, plant, and animal cells [39–41], however the mechanisms behind are unclear. Studies on mitochondria from *Zea mays* L. demonstrate that desiccation increases respiration rates which in turn increases radical formation [40]. Membrane and protein perturbations caused by matric stress (e.g., by PEG-8000 exposure) are likely to impair electron flow leading to increased ROS formation [38, 40, 42]. However, also Fe^2+^ autoxidation induced by membrane lipids in response to desiccation has been suggested as a source of ROS [43].

The most severe consequence of oxidative stress is cell death, which can be caused by lethal damage of proteins, components of the lipid membrane, and nucleic acids [19, 44, 45]. Viability staining of cells subjected to matric stress indicated that mortality was not a major consequence of the increased ROS levels in our study. This was not surprising for cells incubated under −0.5 MPa since accumulation of ROS was not detectable in our system. However, even for cells exposed to extensive matric stress for 46 h the reduction in viability compared to untreated cells was < 15 %. Hence, it is reasonable to state that the cells were subjected to sublethal stress conditions.

We could demonstrate induction of major oxidation stress defense genes by *P. putida* mt-2 under both mild and extensive matric stress. Consequently, the current study adds new knowledge on the physiological responses to accumulation of ROS as a consequence of desiccation. We took advantage of employing a panel of stable GFP-bioreporters based on promoters for genes previously reported to be involved in the response to oxidative stress [20, 46]. The reporters collect the prehistory of promoter activity up till the sampling point, and therefore enable detection of small differences in expression of a fast responding gene. Hereby we were able to detect induction of *katA* and *ahpC*, and also of *osmC*, which is putatively involved in hydroperoxide resistance. Superoxide radicals are eliminated by superoxide dismutase, and significant induction of the corresponding genes has been found in diverse bacterial species following desiccation [31, 41]. However, *sodB* did not appear to be involved in protection against matric stress-derived ROS in *P. putida* mt-2.

Application of the bioreporter panel provides a broad coverage of different oxidative stressors and stress levels. Catalase and alkylhydroperoxide reductase, encoded by *katA* and *ahpCF*, respectively, are both regulated by OxyR. Both functions are known to act as primary scavengers of H_2_O_2_ in *Escherichia coli* and, based on data from this work, they likely play a similar role in *P. putida* [13, 47]. The P_*katA*_ construct had a higher response than P_*ahpC*_ towards accumulation of endogenous ROS detected with the fluorescent ROS-responsive probe H_2_DCF-DA; in particular when facing increased ROS accumulation under extensive matric stress. In accordance, catalases are reported to function better with higher peroxide concentrations [13]. AhpCF functions as a peroxidase that is dependent on NADH to restore its activity, and hence is not effective in removing large amounts of peroxides [48]. This could explain the poorer induction of the *ahpC* reporter under extensive matric stress (−1.5 MPa) than under mild matric stress (−0.5 MPa) after 46 h.

The *osmC* gene encodes an enzyme, which in *E. coli* is referred to as a hydroperoxide resistance protein involved in defense against organic hydroperoxides in particular [15, 49]. In *Pseudomonas aeruginosa*, *osmC* is induced by ethanol and osmotic stress, but not by peroxides, and an *osmC* mutant does not show impaired survival under oxidative stress [50]. In *P. putida*, the exact function of OsmC has not yet been addressed. The current study shows that P_*osmC*_ responds poorly to H_2_O_2_, but the promoter is clearly induced by matric stress. These results suggest that OsmC in *P. putida* mt-2 could be involved in a more general protection against desiccation damage, rather than specifically in defense against the associated production of ROS. Nevertheless, induction of *osmC*, in conjunction with the induction of major oxidative stress defense genes as *katA* and/or *ahpC*, may still be a useful indicator of matric stressed cells challenged by increased ROS levels. In a study by Gülez and coworkers [21], the expression of *osmC* (but none of the classical oxidative stress defense genes) was upregulated in the transcriptome analyzed after 4, 24, and 48 h when *P. putida* KT2440 was challenged with mild matric stress (−0.4 MPa). As the level of mRNA shows rapid temporal dynamics it is possible that expression of these genes were missed in the study by Gülez and coworkers. It should however be noted that these authors used a porous surface model system to impose matric stress as opposed to the PEG-8000 simulated stress employed in the current study and these two stress scenarios appear to lead to expression of slightly different genes [21].

At sublethal levels of ROS accumulation, as those experienced by *P. putida* mt-2 in the current study, oxidative stress-induced DNA damage plays a role in generating adaptive mutations, conferring fitness advantages to cells in challenging environments [26, 51, 52]. Desiccation is reported to induce DNA repair mechanisms such as the MutT system [21, 31, 53], but in *P. putida* mt-2 it remains unknown whether the SOS system, and its error-prone DNA polymerases is activated during matric stress.

Under extensive matric stress we detected induction of the bioreporter harboring the promoter of a resident prophage gene, PP3901. Prophage-induction is a known stress-response in *P. putida* and other bacteria mediated by the SOS system [39, 54–56]. In *P. putida* KT2440, the SOS regulon includes two LexA proteins, and only the gene PP3901, which is required for DNA damage-mediated induction of several other prophage genes, is under control of both proteins [25]. This gene has not been used as an indicator of DNA damage before, but its expression was previously found to be upregulated in mitomycin C-treated cells [25]. As the promoter clearly was induced by DNA-damage introduced by exposure to nalidixic acid, our result indicates that the DNA-damage mediated SOS-response was indeed activated, albeit not very highly, in matric stressed cells. This DNA damage did not translate into a detectably increase in mutation frequency in the genome of *P. putida* mt-2 when exposed to extensive matric stress. Thus we can conclude that neither oxygen radicals themselves, nor error-prone DNA damage repair mechanisms introduced mutations to the desiccated cells despite the high ROS levels detected. Our study therefore provides indications that desiccation stress experienced at matric potentials down to −1.5 MPa, i.e., the permanent wilting point of plants, ensuing production of ROS and the subsequent moderate induction of the SOS system can be managed by *P. putida* mt-2 without starting a genetic diversification regime. In this respect, it is often ignored that induction of stress defenses does not necessarily signifies that a cell *per se* is stressed. In contrast, induction of the defense may provide the cell with tolerance towards the stressor in question. Oxidative stress is only encountered when the level of radicals exceed the capacity of the cell’s defense system to detoxify the oxidants [23]. The concomitant analysis of ROS accumulation, oxidative defense gene expression, viability and response to DNA damage, which is carried out in the current study permit us to make indications of the stress level of desiccated cells. Under mild matric stress, early induction of the defense system of *P. putida* mt-2 seems to be able to eliminate ROS at the same rate as it is produced, at least in the initial part of the experiment. This is supported by the high viability of these cells. Hence these cells cannot be categorized as stressed, although, in the long term the defense might be overwhelmed resulting in ROS accumulation, as we started to observe after 46 h. Contrary, for cells under extensive matric stress, we speculate that ROS formation exceeds the capacity of the defense system already within 12 h with a resulting slight decrease in viability. Nevertheless, the stress was not translated into higher mutation frequencies.

The stress model adopted in the current study captures essential features of the life conditions for *P. putida* mt-2 residing in a drying soil as we used the non-permeating solute PEG-8000 to simulate matric stress to cells in liquid culture. However, it does not reflect in its entirety all constraints to the bacterial cells that they would encounter in a drying soil. For instance, the thin water films on soil surfaces in unsaturated pore spaces reduce the substrate and nutrient availability. Thus, less energy may be available for production of antioxidants, and secondly carbon starvation, which occurs in soil [57], is known to induce mutagenesis through an RpoS-mediated response [10, 11, 20, 58]. In this respect, the panel of hereby described bioreporters will be valuable for examining the manifestation of traits detected in the test tube in a more complex conditions, whether in soil or in the distinct environment of a biotechnological setup.

## Conclusion

Combining *in vivo* detection of endogenous ROS with viability staining and induction-profiling of genes involved in the oxidative stress response revealed that *P. putida* mt-2 is able to restrict major cellular damages from matric stress-induced accumulation of ROS. This is done by inducing the oxidative stress defense system, including both a catalase and peroxidases. Under conditions of mild matric stress, the defense system seems to be able to remove excess ROS, however under extensive matric stress conditions ROS is built up over time. This had nevertheless only a minor impact on total population viability, and despite a slight induction of the SOS response, the accumulation of ROS did not result in increased mutation frequencies in this system. Future work conducted under more realistic conditions in desiccated soil is now needed to determine if these responses can be generalized for bacteria in the environment. The developed panel of bioreporters will also in these more natural systems be a valuable tool.

## Methods

### Bacterial strains and growth conditions

Wild-type strain *Pseudomonas putida* mt-2, obtained from Deutsche Sammlung von Mikroorganismen und Zellkulturen GmbH (DSMZ, DSM-6125), and *P. putida* mt-2 strains carrying plasmid-borne msfGFP-reporter constructs, as well as strains with chromosomal insertions of mini-Tn*7* transposon harboring msfGFP-reporter cassettes were used throughout this study. All strains of *P. putida* mt-2 were cultured in M9 minimal medium (6 g/L Na_2_HPO_4_, 3 g/L KH_2_PO_4_, 0.5 g/L NaCl, 1 g/L NH_4_Cl, 0.13 g/L MgSO_4_, and 0.01 g/L CaCl_2_) containing 10 mM sodium succinate at 28 °C with agitation at 150 r.p.m. For standard cloning and plasmid propagation, *Escherichia coli* CC118λpir and DH5α were used. *E. coli* strains were cultured in Luria-Bertani broth (LB) (10 g/L tryptone, 5 g/L yeast extract, and 10 g/L NaCl) at 37 °C and agitation at 150 r.p.m., or grown on solid LB medium containing 15 g/L agar. When needed, antibiotics were applied to the solid or liquid culture media (10 μg/mL gentamycin or 50 μg/mL kanamycin).

All experiments with *P. putida* mt-2 were conducted as follows. Overnight cultures were pelleted by centrifugation (5000 × *g* for 10 min, 21 °C), and cells were resuspended and diluted in fresh medium to obtain an optical density at 600 nm (OD_600_) of 0.2. In experiments with chemical stress-induction by H_2_O_2_, PQ or nalidixic acid, cultures were incubated for 2.5 h before addition of one of the stressors to final concentrations of 1.5 mM H_2_O_2_, 100 μM PQ, or 30 μg/mL nalidixic acid. Untreated cultures served as controls in all experiments. In experiments with simulation of desiccation by addition of polyethylene glycol (molecular weight = 8000; PEG-8000), the cultures were incubated for 2.5 h and then pelleted by centrifugation as described above before resuspending the cells in M9 medium containing either 150 g/L or 330 g/L PEG-8000 to obtain matric potentials of −0.5 MPa or −1.5 MPa, respectively [8].

### Quantification of expression of genes involved in oxidative stress defense by RT-qPCR

Wild-type mt-2 cells were incubated in the presence of oxidative stress generators (H_2_O_2_ and PQ), DNA-damage generator (nalidixic acid) or without a stressor as described above. After 30 min and 2 h of incubation, 100 μL samples were collected, immediately flash frozen in liquid nitrogen and stored at −70 °C. Before nucleic acid extraction, samples were treated with lysozyme (100 μL of 1 mg/mL in 10 mM Tris · HCl buffer, pH 8, per sample of 100 μL) for 20 min at room temperature. DNA and RNA were co-extracted with the AllPrep DNA/RNA extraction kit (QIAGEN, UK) according to the manufacturer’s protocol. Prior to reverse transcription of RNA, genomic DNA was eliminated from the RNA samples with RQ1 RNase-free DNase 1 (Promega, USA) according to the manufacturer’s protocol. cDNA was synthesized immediately thereafter by using the Omniscript RT Kit (QIAGEN) including random hexamer primers (Promega) and SUPER RNase inhibitor (Ambion, USA) as previously described [59]. DNase-treated control reactions were prepared in parallel for RNA samples without addition of reverse transcriptase to ensure the absence of genomic DNA contamination.

Expression of the genes *katA* (PP0481), *ahpC* (PP2439), *sodB* (PP0915), *osmC* (PP0089), and the prophage gene PP3901 was quantified by qPCR using the Stratagene Brilliant III SYBR Green QPCR Master Mix (Agilent Technologies, USA). The primers used for amplification were the following: katA-Fw (5′-CTTGAAGACCGAAATGGAG-3′) and katA-Rv (5′-GTTTGTTTACCGACCTCTTC-3′) [46] for *katA,* ahpC-Fw (5′-GCGTGGAAATCTACGGTGTT-3′) and ahpC-Rv (5′-CTTCGCCTTCTTTCCACTTG-3′) for *ahpC*, sodB-Fw (5′-AGGGAACCGTCAGCTTTCTT-3′) and sodB-Rv (5′-GCACCACAACACCTATGTCG-3′) for *sodB*, osmC-Fw (5′-GCCTGAAGGATGGAAAAGGT-3′) and osmC-Rv (5′-AAAACCGTCAGCTTGCTTGT-3′) for *osmC*, and PP3901-Fw (5′-CACATCCTTCGACCTCCTTG-3′) and PP3901-Rv (5′-TTTTCCCAGGTCACACGAAC-3′) for the PP3901 gene. A standard curve was prepared as a tenfold serial dilution of DNA extracted from a stationary phase culture of *P. putida* mt-2. Gene expression was calculated as mRNA transcripts of each gene, determined from the numbers of cDNA copies, normalized to the DNA copy number of the corresponding gene.

### Construction of GFP-based bioreporters

The oxidative stress-responsive bioreporters were constructed by inserting the promoter regions of *katA*, *ahpC*, *sodB*, and *osmC* into *Eco*RI/*Bam*HI sites of the pBBR1-based broad-range-host vector pSEVA237M harboring a promoterless monomeric superfolder *gfp* gene [36]. The DNA-damage-inducible bioreporter was constructed by inserting the promoter region of the PP3901 gene into *Eco*RI/*Bam*HI sites placed upstream a promoterless superfolder *gfp* gene [60] of a mini-Tn*7*-based delivery vector (Víctor de Lorenzo lab collection).

Fragments including the promoter regions of the genes were amplified by PCR with the following primers (restriction enzyme recognition sites are shown in italics): katA-EcoRI-Fw (5′-CGAT*GAATTC*GGAAGACAGCGTTGCTAACC-3′) and katA-BamHI-Rv (5′-CGAT*GGATCC*TCCATTTCGGTCTTCAAGG-3′) for the region −391 to −7 upstream of *katA*, ahpC-EcoRI-Fw (5′-CGAT*GAATTC*GAGCCCCTCCTCCTTGAAT-3′) and ahpC-BamHI-Rv (5′-CGAT*GGATCC*TCATGGGGTTGGAATCAGTT-3′) for the region −321 to −14 upstream of *ahpC*, osmC-EcoRI-Fw (5′-CGAT*GAATTC*GCCACCCAGAAGCGGTTA-3′) and *osmC*-*Bam*HI-Rv (5′-CGAT*GGATCC*GATGCCTCCTGGTCACTG-3′) for the region −295 to −1 upstream of *osmC*, and PP3901-*Eco*RI-Fw (5′-CGTA*GAATTC*GGCTCCTTACCTGCGACTAA-3′) and PP3901-*Bam*HI-Rv (5′-CGTA*GGATCC*GGTGGTAGCGCTCCATTTAC-3′) for the region −146 to −1 upstream of PP3901. Standard molecular cloning procedures were used to construct the pSEVA237M-based reporter plasmids or the mini-Tn*7* vector. The correct orientation and sequence of the promoters were verified with PCR and sequencing of a 1-kb region including the corresponding promoter and part of the msf-*gfp* gene. Reporter and mini-Tn*7* plasmids were introduced by electroporation into *E. coli* DH5α or CC118λpir, respectively, and then into wild-type *P. putida* mt-2. The plasmid pUX-BF13, providing the transposase genes, was used for chromosomal Tn*7* integration [61]. Finally, the correct placement and orientation of the Tn*7* insertion was verified by PCR as described previously [62].

### Detection of bioreporter responses, intracellular ROS, and cell viability by flow cytometry

Flow cytometry analysis was performed using a BD FACSCalibur flow cytometer (Becton Dickinson, CA) equipped with an argon-ion laser of 15 mW with excitation at 488 nm. To detect induction of bioreporters under the tested conditions, cells were first gated in a side scatter (SSC) vs. forward scatter (FSC) plot, and then fluorescence from GFP was recorded in the FL 1 channel (515–545 nm). For fluorescence detection culture samples of 0.5 ml were pelleted by centrifugation (7000 × *g*, 5 min, 21 °C) and re-suspended in sterile-filtered 0.9 % (w/v) NaCl to obtain a similar optical density at 600 nm (OD_600_).

Detection of cells accumulating intracellular ROS after exposure to PEG-8000 was carried out using the ROS-sensitive green fluorescent dye 2′,7′-dichlorodihydrofluorescein diacetate (H_2_DCF-DA [Sigma-Aldrich Co.]). Cells incubated with 1.5 mM H_2_O_2_ were included as a positive control (data not shown). For each mL of cells re-suspended in 0.9 % (w/v) NaCl, 20 μL of 1 mg/mL solution of H_2_DCF-DA in DMSO was added and the samples were incubated in the dark for 30 min at room temperature. Green fluorescence emission from ROS-dependent oxidation of H_2_DCF-DA was detected with the FL1 detector after gating the cells in a SSC vs. FSC plot.

Viability of cells following exposure to PEG-8000-induced matric stress was determined by flow cytometry essentially as described by DeRoy et al. [63]. Cells were incubated in the dark with 10 μL/mL EDTA (500 mM, pH 8) and 10 μL/mL staining solution (400 μM propidium iodide in DMSO from the BacLight Kit [Invitrogen], and 100× SYBR Green I in DMSO [Invitrogen]) for 30 min. Cells with compromised membranes were distinguished from cells with intact membranes in a plot of green fluorescence from SYBR Green collected by the FL1 detector vs. red fluorescence collected by the FL3 detector (675–715 nm).

Data from at least 50,000 cells were collected from each sample and analyzed in the software Cyflogic™ 1.2.1 (CyFlo Ltd.) for all flow cytometry experiments.

### Frequency of rifampicin resistance mutations

After 72 h of incubation in the presence of PEG-8000 (150 g/L and 330 g/L), H_2_O_2_ (1.5 mM), or without any stressor, cultures were pelleted by centrifugation (5000 × *g*, 10 min, 21^0^ C), and cells were resuspended in 0.9 % (w/v) NaCl. From each triplicate culture, 100 μL of the cell suspension were spread in duplicate on LB agar plates containing 100 μg/mL rifampicin. For each harvested culture, six 10-μL droplets of 10^−5^ and 10^−6^ dilutions in 0.9 % (w/v) NaCl were spotted on LB plates. The number of Rif^R^ colonies was counted after 48 h of incubation at 28 °C and normalized to the number of colonies on LB control plates giving the Rif-mutation frequency per CFU for each condition tested.

### Statistical analysis

Mean values of two data sets were compared with the Student’s *t*-test with *P* < 0.05 as the cut-off value for statistical significance. All experiments were comprised of three biological replicates and carried out at least twice.

## Abbreviations

H_2_DCF-DA: 2′,7′-dichlorodihydrofluorescein diacetate
PEG-8000: Polyethylene glycol with molecular weight 8000
PQ: Paraquat
ROS: Reactive oxygen species
